# Root exudates facilitate the regulation of soil microbial community function in the genus *Haloxylon*

**DOI:** 10.3389/fpls.2024.1461893

**Published:** 2024-09-19

**Authors:** Deyan Wu, Xuemin He, Lamei Jiang, Wenjing Li, Hengfang Wang, Guanghui Lv

**Affiliations:** ^1^ College of Ecology and Environment, Xinjiang University, Urumqi, Xinjiang, China; ^2^ Key Laboratory of Oasis Ecology of Ministry of Education, Xinjiang University, Urumqi, Xinjiang, China; ^3^ College of Life Science, Xinjiang Agricultural University, Urumqi, Xinjiang, China; ^4^ Key Laboratory for Ecological Adaptation and Evolution of Extreme Environment Biology, Xinjiang Agricultural University, Urumqi, Xinjiang, China

**Keywords:** root exudates, genus *Haloxylon*, soil microorganisms, root traits, rhizosphere

## Abstract

**Introduction:**

Root exudates act as the "language" of plant-soil communication, facilitating crucial interactions, information exchange, and energy transfer between plants and soil. The interactions facilitated by root exudates between plants and microorganisms in the rhizosphere are crucial for nutrient uptake and stress resilience in plants. However, the mechanism underlying the interaction between root exudates and rhizosphere microorganisms in desert plants under drought conditions remains unclear, especially among closely related species.

**Methods:**

To reveal the ecological strategies employed by the genus Haloxylon in different habitats. Using DNA extraction and sequencing and UPLC-Q-Tof/MS methods, we studied root exudates and soil microorganisms from two closely related species, *Haloxylon ammodendron (HA)* and *Haloxylon persicum (HP)*, to assess differences in their root exudates, soil microbial composition, and interactions.

**Results:**

Significant differences were found in soil properties and root traits between the two species, among which soil water content (SWC) and soil organic carbon (SOC) in rhizosphere and bulk soils (*P* < 0.05). While the metabolite classification of root exudates was similar, their components varied, with terpenoids being the main differential metabolites. Soil microbial structure and diversity also exhibited significant differences, with distinct key species in the network and differential functional processes mainly related to nitrogen and carbon cycles. Strong correlations were observed between root exudate-mediated root traits, soil microorganisms, and soil properties, although the complex interactions differed between the two closely relative species. The primary metabolites found in the network of *HA* include sugars and fatty acids, while *HP* relies on secondary metabolites, steroids and terpenoids.

**Discussion:**

These findings suggest that root exudates are key in shaping rhizosphere microbial communities, increasing microbial functionality, fostering symbiotic relationships with hosts, and bolstering the resilience of plants to environmental stress.

## 1 Introduction

Rhizosphere refers to the narrow region of soil that surrounds and is influenced by plant roots, where various interactions between roots and microorganisms that inhabit their root vicinity, and high concentrations of plant-derived organic exudates and mucilage (Korenblum et al., 2020; Vieira et al., 2020). Root exudates, serving as the “language” of rhizosphere communication, are pivotal in facilitating interactions, information exchange, and energy transfer between plants and soil (Venturi and Keel, 2016; Zhao et al., 2021). Root exudates comprise various compounds, such as amino acids, organic acids and other secondary metabolites, which are vital for plant functional metabolic pathways, with their composition affecting the physicochemical properties and nutrient availability of rhizosphere soil (Bais et al., 2006; Zhalnina et al., 2018).

Soil microorganisms are crucial components of the soil ecosystem, playing a vital role in maintaining ecological balance and nutrient cycling (Cui et al., 2018; Sahu et al., 2019). Root exudates as a medium for the co-evolution of plants and microorganisms (Morgan et al., 2005), they extend the functional traits of plants by participating in a series of processes, including nutrient acquisition, growth promotion, and enhancing the ability to resist environmental stress (Bai et al., 2022). Root exudates stimulate microbial colonization at the root-rhizosphere soil interface, affecting the relative abundance and activity of soil microorganisms (Yin et al., 2013; Ribbons et al., 2016). This process enhances resource acquisition and adaptation to adverse environments (Haichar et al., 2014), such as drought and salinization, representing the evolutionary response of plants to external environment changes (Yin et al., 2013). The preference of soil microorganisms for root exudates drives the assembly of rhizosphere microbial communities (Zhalnina et al., 2018), allowing plants to selectively promote beneficial microorganisms (Bulgarelli et al., 2015), while deterring pathogenic or detrimental ones (Venturi and Keel, 2016). A previous study found that *Arabidopsis* and *Ageratina adenophora* could recruit beneficial bacteria by releasing specific compounds from root exudates to promote growth (Harbort et al., 2020; Sun et al., 2021). Root exudates facilitate plant‐microbial community interactions that affect plant growth and adaptation, fostering mutualistic relationships or antagonizing soil microorganisms to affect plant growth (Eppinga et al., 2006; Kardol et al., 2007).

Root exudates play an important role in interspecies chemical communication, acting as chemical signals between plant roots and other soil organisms (including the roots of neighboring plants), and inducing changes in root behavior (Bais et al., 2006; Semchenko et al., 2014; Xia et al., 2019). Plants can modify soil pH through changes in root exudate composition, which affects pathogen and beneficial microbial colonization (Lareen et al., 2016). Manipulating plant traits to enhance plant-microbial interactions that increase plant resilience to adversity and optimize soil nutrient cycling (De Vries et al., 2020). These interactions are crucial for maintaining and stabilizing ecosystem functions. For example, plant root traits and the composition of rhizosphere microbial communities promote plant yields (Lareen et al., 2016). These traits and exudates adjust in response to resource competition among species (Meier et al., 2020; Wen et al., 2019) and they are intricately linked to resource acquisition and utilization functions, such as the contents of roots N and P in roots (Cui et al., 2018; Sun et al., 2021). Hence, further observation is needed to assess the relationship between these rhizosphere microbes and plant community dynamics mediated by plant traits (Chai et al., 2019). Moreover, rhizosphere microbes play a crucial role in fostering host phenotypic plasticity, aiding host plant adaptation to environmental conditions (De La Fuente Cantó et al., 2020). However, studies on the role and regulation of root exudates in the rhizosphere environment of desert soils remain relatively few, which are affected by various factors and conditions, especially congenerics plants. Therefore, the mechanism underlying the interaction between exudates and their rhizosphere microorganisms in closely related species under drought conditions remains unclear.

Root exudation mediated plant-soil microbial interactions are crucial in facilitating active plant adaptation to microenvironment and stress resistance (De Vries et al., 2020). These exudates modify the soil properties to help plants withstand the stress of adversity (Baetz and Martinoia, 2014; Zhu et al., 2009). In the Gurbantunggut Desert, two closely related species, *Haloxylon ammodendron* (C. A. Mey.) Bunge, primarily inhabiting on the plain saline-alkali land, and *Haloxylon persicum* Bge. ex Boiss. et Buhse, grows on the sand soil of the dunes with a distinct species distribution mode (Song et al., 2006). Studies have explored environmental factors as potential causes for this pattern but have not reached a consensus. This study focuses on root exudates and investigates root characteristics, exudates, soil environmental factors, rhizosphere microorganisms, and their interactions. It analyzed root exudates composition, soil microbial community structure variations, and the interplay among environmental factors, plant traits, and rhizosphere microorganisms. Therefore, this study aims to elucidate the ecological adaptation strategies employed by the genus *Haloxylon* in different habitats. The findings may help elucidate the underlying mechanism and distribution pattern of desert plants adapting to harsh environments. It also seeks to provide valuable insights that can greatly contribute to desertification control and ecological restoration efforts.

## 2 Materials and methods

### 2.1 Study site

The research site was situated in the Ebinur Lake Wetland National Nature Reserve, Xinjiang (83°32′–83°35′E, 44°35′–44°42′N). In this region, the average annual precipitation was approximately 100 mm, with annual evaporation rates >1600 mm, an average annual temperature range of 6–8°C and approximately 2800 h of sunshine annually (Wang et al., 2022). The long-term scarcity of precipitation resulted in severe drought stress in the local ecosystem (Li et al., 2022a). The soil is predominantly gray desert soil, aeolian sandy, meadow soil, and swamp soils (Wang et al., 2022). The local zonal vegetation consisted of sparse, extreme xeromorphic shrubbery, and dominant shrubs in this ecosystem included *H. ammodendron* (*HA*)*, H. persicum* (*HP*), *Tamarix ramosissima, Nitraria schoberi, Reaumuria soongorica*, and *Halimodendron halodendron.*

### 2.2 Sample collection

Based on previous investigation and research in the study area, *HA* is primarily distributed on the saline-alkali land near the river bank of the Akikesu River, and *HP* grows on the semi-fixed dune far away from the river bank. In July 22 to 25, 2021, we chose four sample sites of *HA* approximately 0.5 km from the riverbank towards the Akikesu River, and four sample sites of *HP* approximately 3.5 km from the riverbank, matching in height and size, to collect plant root exudates, and rhizosphere and bulk soil samples (**Supplementary Figure S1**).

Rhizosphere and bulk soil samples were collected from four *HA* and *HP*, respectively. The plants selected from each site of *HA* or *HP* were collected from depth of 0 ~ 40 cm,1 ~ 2 m away from the trunk. All roots at each site are placed in sterile bags to prepare rhizosphere soil samples. In addition, four bare soil samples were collected on unvegetated ground about 10 m away from the plants, at a depth of about 0-40 cm, and these samples were uniformly mixed to form bulk soil samples.

Each root of the plant, measuring approximately 20 cm in length, was selected from the four cardinal directions, ensuring the roots remained undamaged. The soil clinging to the root surface was carefully rinsed off with distilled water. Following a modified version of the *in situ* sample collection method (Phillips et al., 2018), the primary steps were as follows: the cleaned root was placed in a sterile bag filled with sterile glass sand (1 mm particle size) as the culture medium. Simultaneously, a carbon-free nutrient solution (Jakoby et al., 2020) was injected to provide essential nutrients for healthy root development. The opening of the bag was sealed to prevent impurities, and then the aseptic bag, which was wrapped in tin foil, was covered with soil. After 24 h of root recovery, the growth medium was washed with the nutrient solution to collect the exudates, combining all samples from each tree.

After extraction, 16 roots were randomly selected from the roots of both *HA* and *HP*. Their surface moisture was adsorbed using filter paper. The roots were then scanned. Following that, they were analyzed using WinRHIZO software. Subsequently, the scanned roots underwent drying at 65°C for 48 h and then weighed. Follow the methods used in previous studies (Li et al., 2022a), Rhizosphere soil samples were collected by adding a Phosphate Buffered Saline (PBS) solution into the sterile, aseptic bag containing roots. The solution underwent a rinsing process, during which turbidity was collected and subsequently centrifuged. After removing the supernatant, the precipitated material was used as the rhizosphere soil and stored at −20°C until measurement. **Supplementary Table S1** depicts the soil properties of the samples, which were evaluated using soil measurement methods.

### 2.3 DNA extraction, high-throughput sequencing and processing

Total soil genomic DNA was extracted from soil using the MoBio Power Soil DNA Isolation Kit (MoBio Laboratories, Carlsbad, CA, USA).The bacterial PCR amplification employed primers 515F (5’ - GTGCCAGCMGCCGCGG - 3’) and 907R (5’ - CCGTCAATTCMTTTRAGTTT -3’) (Christner et al., 2001), while the fungal PCR amplification used primers ITS_1_F (5’ - CTTGGTCATTTAGAGGAAGTAA - 3’) and ITS2R (5’ - GCTGCGTTCTTCATCGATGC - 3’) (Gardes and Bruns, 1993). PCR amplification was conducted in a 20 μL reaction system using TransGen AP221-02. The PCR products were detected on 2% agarose gel electrophoresis and QuantiFluor was used for quantitative detection ™-ST blue fluorescence quantification system. A cDNA library was constructed on the Illumina MiSeq platform (Illumina San Diego, USA) and high-throughput sequencing (MiSeq PE300) of the PCR amplified fragments was performed.

Utilizing the UPARSE platform version 7.1, gene sequence clustering and elimination of chimeras were executed to achieve 97% ASV convergence towards taxa. The annotation and Bayesian algorithms, and RDP classifier were used for the species classification analysis. After flattening, 2,201 bacterial ASVs (23,444 sequence reads) and 634 fungal ASVs (2,783 sequence reads) were obtained.

### 2.4 Determination of root exudates via UPLC-Q-Tof/MS

The *HA* and *HP* exudates were concentrated to approximately 5 mL using a Rotary evaporator at 45°C and 120 r/min. They were then transferred to a low‐temperature freeze dryer until they became solid powder. Subsequently, 1.5 mL of 70% methanol aqueous solution was added for re-dissolution, followed by a 30-min ultrasound treatment. After centrifugation at 10000 rpm/min for 5 min, 200 μL of the solution was taken and placed into a 2 mL volumetric bottle, then passed through a 0.22-μm filter membrane for use. The samples were separated using a Waters Acuity H-Class ultra-high performance liquid chromatograph (FTN automatic sampler, four-element liquid chromatography pump) and ACQUITY UPLC^®^ BEH C18 column (1.7 μm, 2.1 mm×100 mm) at a temperature of 45°C. The flow rate was maintained at 0.4 mL/min with an injection volume of 2.0 μL.

The MS^E^ mode was employed for data collection, capturing mass spectrum data in positive ion and sensitivity modes. Data storage utilized the centroid mode, and a real‐time mass number correction was achieved using a leucine enkephalin (LE) standard solution, with a 5 μL/min flow rate. Mass Lynx v4.1 software was employed to facilitate MS data collection, which was then imported into UNIFI for peak identification and chemical composition retrieved through the Natural Products database. The obtained products were compared with the KEGG database, followed by metabolite classification and enrichment analyses of the root exudates.

### 2.5 Statistical analysis

Root traits and soil properties were expressed as mean ± standard error (SD). Before statistical analysis of the data, the “shapiro.test” and “Bartlet.test” functions were used to test the normality and variance homogeneity of the data, respectively. If the data do not conform to the normal distribution, or the variance between the groups was not uniform, the “kruskal.test” function was used for non-parametric tests. Metabolites from root exudates were classified using the KEGG database, with classification statistics conducted using the *reshape2* package. Pathway enrichment analysis was conducted using Metabo Analyst 6.0. Orthogonal partial least squares discriminant analysis (OPLS-DA) was used to differentiate intergroup variations in the root exudates of both species, and the variable importance in projection (VIP) of OPLS-DA was calculated. Root exudates that satisfy both criteria of |log2FC| > 1.5 and a t-test with a *P*-value < 0.05 were selected for statistical analysis as metabolites exhibiting significant differences.

The bacterial and fungal network at the family level was constructed based on the Pearman correlation matrix. ASV units with abundance < 20 were excluded, and only correlations satisfying the conditions (r > 0.8 and *P* < 0.05) were considered, and the degree of a node in the network determines its criticality. The network was visualized, and its topological properties were obtained using Gephi (v 0.9.2). Differential bacteria and fungi communities’ analysis were conducted using *ggpubr* package.

The *devtools* and *linkET* packages were utilized for correlation and Mantel analyses involving root traits, environmental factors, root exudates, rhizosphere soil bacteria, and fungi. Node and edge data were obtained using the *igraph* package. Cytoscape (v 3.9.1) facilitated the prediction of root exudates and microbial functional pathways, along with network visualization of soil properties and root traits (r > 0.8 and *P* < 0.05).

All statistical analyses were conducted using R 4.1.3 (http://www.r-project.org/), with the *ggplot2* package used for mapping in statistical analyses (http://www.r-project.org/).

## 3 Results

### 3.1 Difference analysis of root traits and soil properties of genus *Haloxylon*

Significant differences were observed in soil pH, soil water content (SWC), soil salt content (SA), soil organic carbon (SOC), soil total phosphorus (TP), soil available phosphorus (AP), and soil total nitrogen (TN) between *HA* and *HP* treatments (*P* < 0.05). However, no significant differences were observed in soil nitrate nitrogen (NN) (*P* > 0.05). SWC and SOC exhibited significant differences between the rhizosphere and bulk soil of the two species, whereas TN and soil ammonium nitrogen (AN) showed significant differences in the soil of the two species (*P* < 0.05) (**Table 1**).

**Table 1:** Soil property comparison of soil from genus *Haloxylon*.

Soil properties	Unit	HAR	HAB	HPR	HPB
pH	–	8.24 ± 0.20Aa	8.01 ± 0.30Aa	7.29 ± 0.14Ab	7.24 ± 0.18Ab
SWC	%	13.53 ± 1.56Aa	5.31 ± 0.17Ba	1.03 ± 0.11Ab	0.19 ± 0.00Bb
SA	g.kg^-1^	7.59 ± 2.17Aa	4.80 ± 0.90Aa	1.12 ± 0.07Ab	1.00 ± 0.13Ab
SOC	g.kg^-1^	10.84 ± 4.59Aa	4.67 ± 1.37Ba	1.12 ± 0.16Bb	1.38 ± 0.08Ab
TN	g.kg^-1^	1.89 ± 0.72Aa	1.56 ± 0.68Aa	0.76 ± 0.10Ab	0.55 ± 0.13Bb
AN	mg.kg^-1^	3.77 ± 1.73Aa	1.86 ± 0.61Aa	1.28 ± 0.17Aa	0.67 ± 0.17Bb
NN	mg.kg^-1^	11.43 ± 5.72Aa	6.27 ± 2.75Aa	2.70 ± 0.25Aa	3.05 ± 0.33Aa
TP	g.kg^-1^	1.32 ± 0.22Aa	1.37 ± 0.34Aa	0.65 ± 0.04Ab	0.69 ± 0.02Ab
AP	mg.kg^-1^	43.61 ± 19.17Aa	33.98 ± 14.58Aa	9.63 ± 0.77Ab	8.09 ± 1.80Ab

Different capital letters indicate significant differences between the rhizosphere and bulk soil, while the same capital letters denote insignificant differences. Different lowercase letters indicate significant differences between the rhizosphere soils of the genus Haloxylon, or between their bulk soil, whereas the same lowercase letters denote no significant differences between them. HAR and HPR represent the rhizosphere soil of *HA* and *HP*, HAB and HPB represent the bulk soil of *HA* and *HP*. SOC, Soil organic carbon; SA, soil salt content; TN, soil total nitrogen; TP, soil total phosphorus; AN, Soil ammonium nitrogen; NN, soil nitrate nitrogen; AP, Soil available phosphorus.

**Figure 1:**
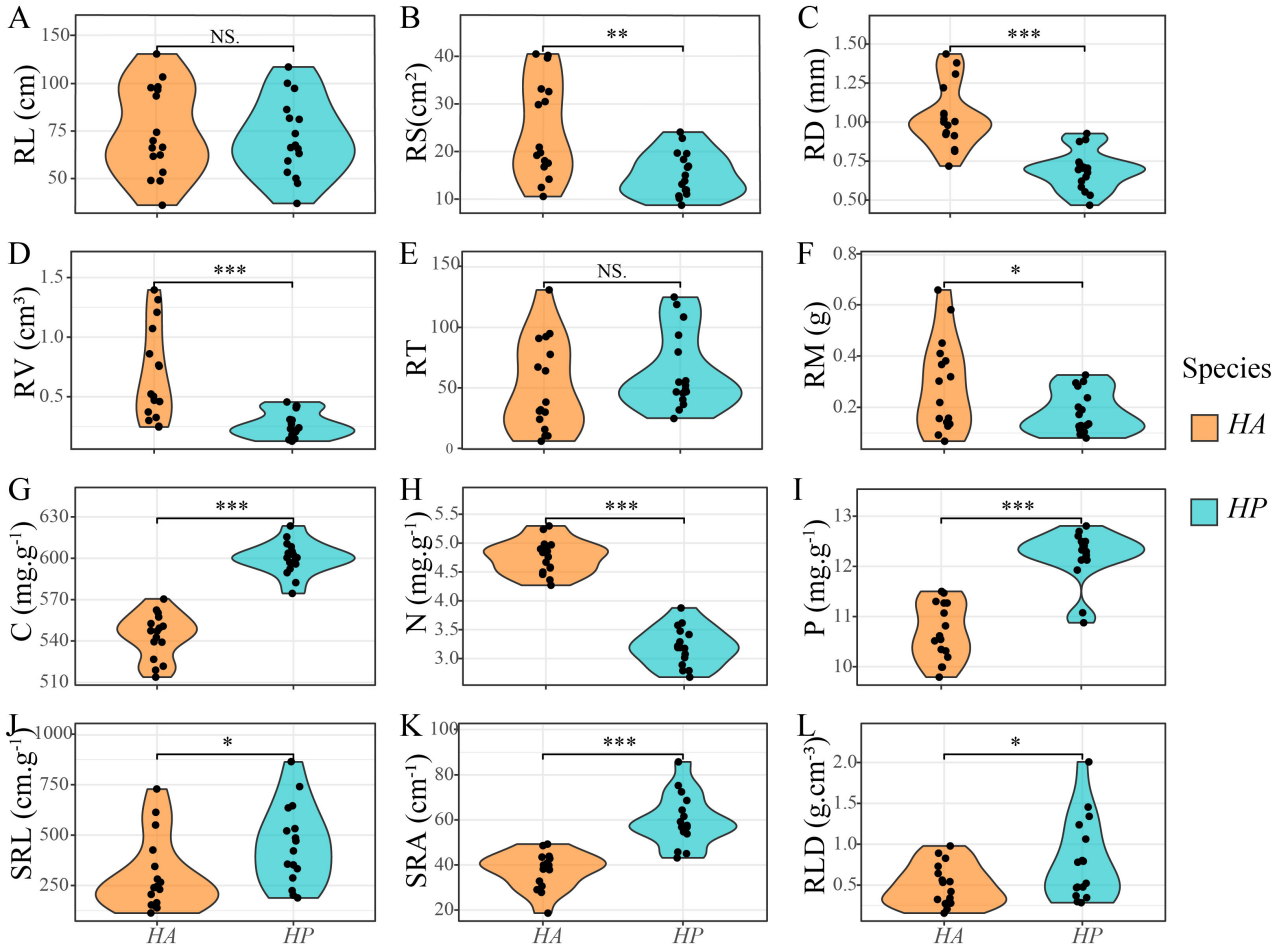
Differences in root traits between *Haloxylon* species. **HA** represent **H. ammodendron**, and *HP* represent *H. persicum*. **(A)** RL, root length; **(B)** RS, root surface area; **(C)** RD, average root diameter; **(D)** RV, root volume; **(E)** RT, number of root tips; **(F)** RM, dry weight of root biomass; **(G)** C, root carbon content; **(H)** N, root nitrogen content; **(I)** P, root phosphorus content; **(J)** SRL, specific root length; **(K)** SRA, specific surface area; **(L)** RLD, root tissue density. *, **, *** indicated significant levels of *P* < 0.05, *P* < 0.01, and *P* < 0.001 respectively, while NS indicated no significant level.

Significant differences were observed in root characteristics between *HA* and *HP* treatments (**Figure 1**). The root surface area (RS), mean root diameter (RD), root volume (RV), and nitrogen (N) content of *HA* were significantly higher than those of *HP* (*P* < 0.01). In contrast, carbon (C) and phosphorus (P) contents, specific root length (SRL), specific surface area (SRA), and root tissue density (RLD) of *HP* were significantly higher than those of *HA* (*P* < 0.05).

### 3.2 Composition and difference analysis of root exudates of genus *Haloxylon*

**Figure 2:**
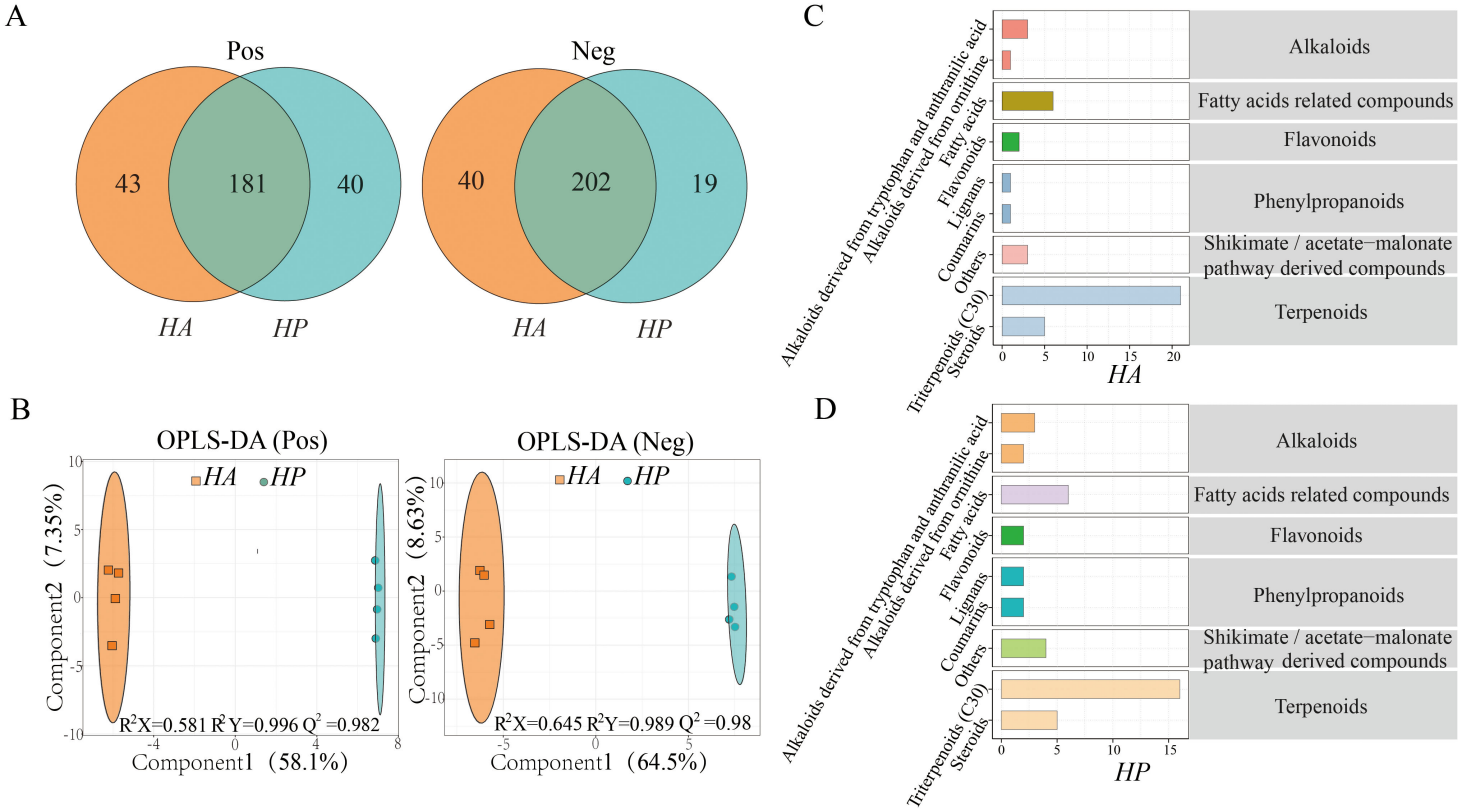
Comparison of root exudate composition in the genus *Haloxylom.* **(A)**, the root exudate composition. **(B)**, the OPLS-DA of the root exudates. **(C, D)** illustrate the root exudate compositions of the two species in the KEGG database.

By chemical composition analysis, 263 and 261 positive and negative ion compounds were identified in the root secretions of *HA* and *HP* (**Figure 2A**), and the common compounds accounted for 68.8% and 77.4%, respectively. These compounds were compared using the KEGG database, and 85 positive and 83 negative ion metabolites were identified. The OPLS-DA analysis of metabolites accounted for 65.45% and 73.13% of the variance, respectively (**Figure 2B**). The predictive power of the positive and negative models was high at 0.982 and 0.98(Q^2^Y>0.9, R^2^Y>Q^2^Y), respectively. This indicated a significant difference in metabolites between the root exudates of *HA* and *HP*.

According to the Phytochemical compounds in the KEGG compounds, the metabolites identified in the root exudates of the two species were categorized mainly into alkaloids, fatty acid-related compounds, flavonoids, phenylpropanoids, terpenoids, and shikimate/acetate malonate pathway-derived compounds. Among these, triterpenoids were the most abundant, followed by steroids and fatty acids. The classification of KEGG metabolite compounds, metabolite pathways, and enrichment were comparable to the root exudates of both species (**Figures 2C, D**; **Supplementary Figure S2**).

**Figure 3:**
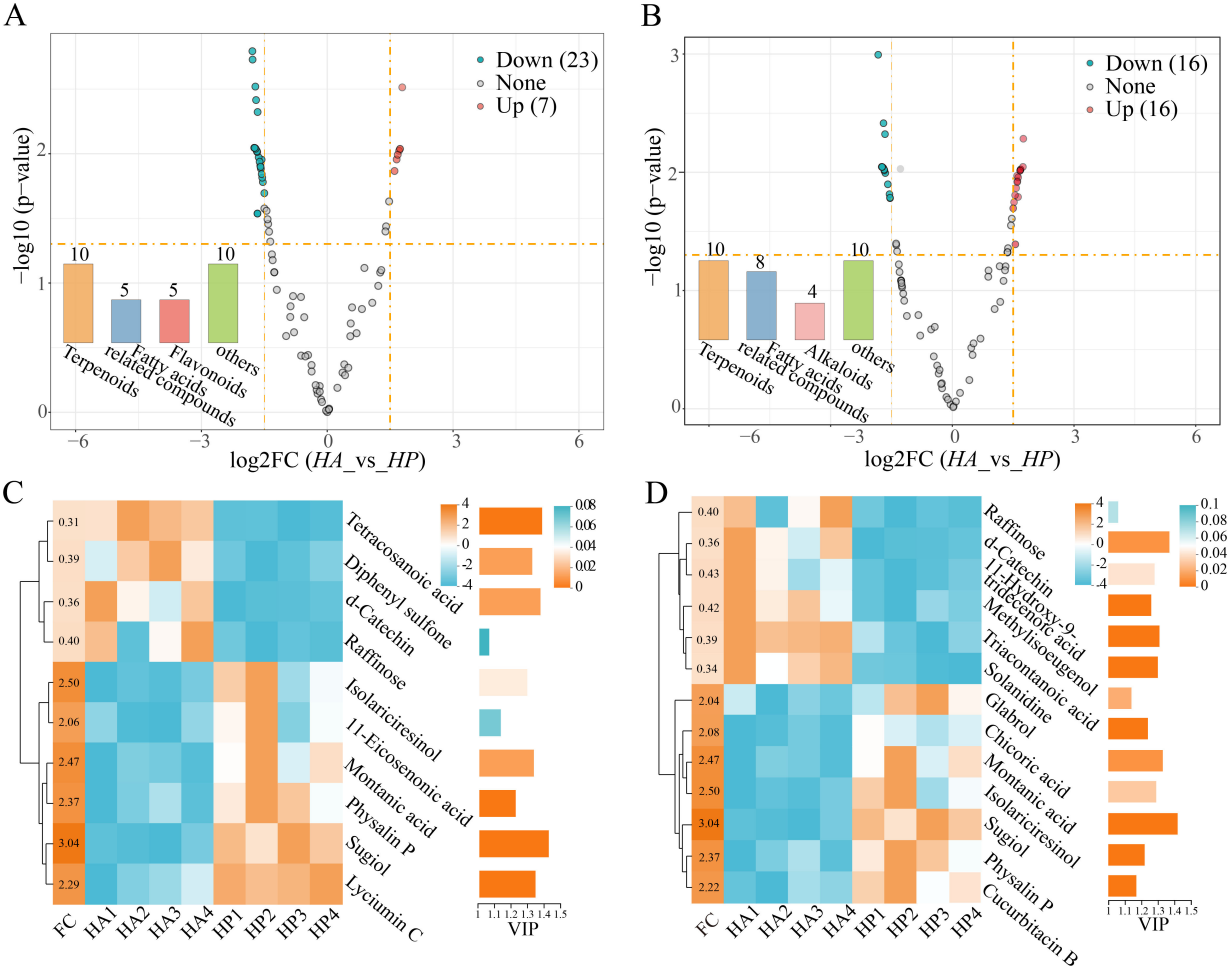
Difference in root exudates within the genus *Haloxylon.* **(A, B)** represent the volcano plot, with exudates that increased and decreased twofold, as shown in orange and blue, respectively, while other metabolites are depicted in gray. **(C, D)** displayed the Heatmap cluster and VIP analysis of the differential exudates between the two species. **(A, C)** are the positive ion metabolites. **(B, D)** are the negative ion metabolites of exudates.

Based on the OPLS-DA analysis (|log2FC|>1.5, *P*<0.05), 622 metabolites were identified between *HA* and *HP*, comprising 30 positive and 32 negative ions. Among these, 23 metabolites in *HP* exhibited higher content (7 positive and 16 negative ions), while 39 metabolites (23 cations and 16 anions) showed higher content in *HA* (**Figures 3A, B**). The significantly different metabolites in the root exudates of the two species were further analyzed using clustering heat maps and VIP bar graphs (FC > 2 or FC < 0.5, VIP > 1) (**Figures 3C, D**). Terpenoid compounds emerged as the primary differential metabolites in the root exudates of the two species, with fatty acid-related compounds showing higher levels in *HP* than those of *HA.*

### 3.3 Analysis of soil microbial community of genus *Haloxylon*

**Figure 4:**
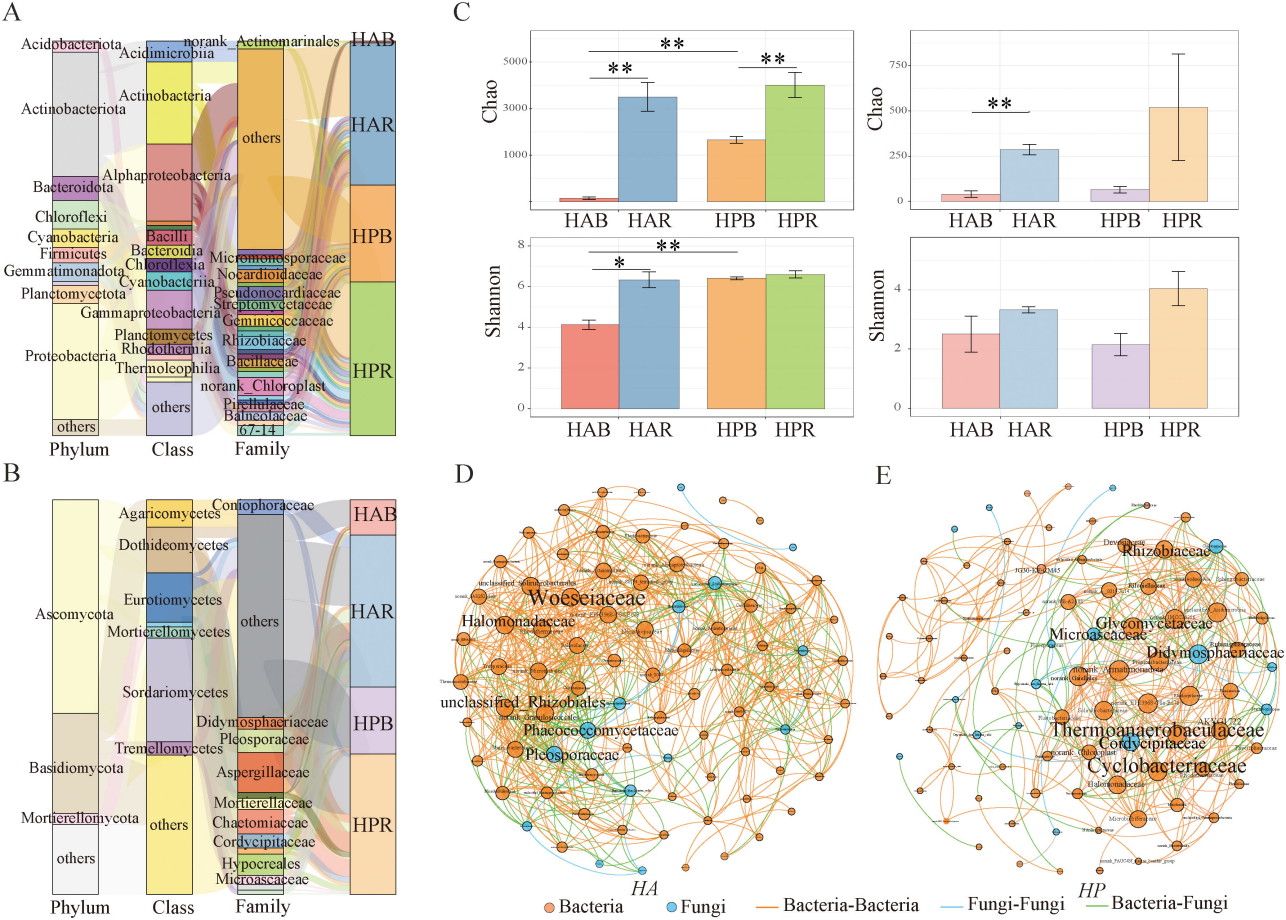
Soil bacterial and fungal communities of genus *Haloxylon.* **(A, B)** show the composition of soil bacterial and fungal communities at the phylum level. **(C)** displays the soil microbial diversity comparison between *HA* and *HP*, indicated by *P < 0.05, **P < 0.01. **(D)** depicts the networks of soil microbial communities at the family level for *HA*, and **(E)** is the networks for *HP*, with the circle size representing the number of degrees. HAR and HAB refer to the rhizosphere and bulk soil of *HA*, respectively, while HPR and HPB denote the rhizosphere and bulk soil of *HP*, respectively.

At the phylum level, 11 phyla with relative abundances exceeding 1% were identified, with Actinobacteria (31.53%) and Proteobacteria (29.36%) emerging as dominant bacteria. Among the dominant fungi, Ascomycota (54.15%) and Basidiomycota (25.34%) were the dominant groups (**Figures 4A, B**), and the community structure displayed significant differences (**Supplementary Figure S3**). Regarding soil bacteria diversity, significant differences were observed in the Chao and Shannon indices for *HA* (*P* < 0.05), along with a significant difference in the Chao index for *HP* (*P* < 0.05). Significant differences were observed in the Chao and Shannon indices of soil bacteria between bulk *HA* and *HP* samples (*P* < 0.01). However, no significant difference (*P* > 0.05) was observed in the rhizosphere soil bacterial diversity index between the two plants. Regarding the soil fungi diversity, the Chao index showed a significant difference (*P* < 0.01), while the Shannon diversity index did not exhibit a significant difference (*P* > 0.05) (**Figure 4C**). Furthermore, the rhizosphere fungal diversity surpassed that of the bulk soil. Additionally, differences were observed in the species of soil bacteria and fungi within the *Haloxylon* genus (**Supplementary Figure S4**).

The network association between soil bacteria and fungi was analyzed using Spearman’s correlation (|r|>0.8, *P*<0.05) (**Figures 4D, E**; **Supplementary Table S3**). The numbers and edge interaction ratios within the bacterial and fungal interaction networks of both species were similar. However, the co‐occurrence network of *HA* exhibited a higher gram density, average degree, and average clustering coefficient than that of *HP* while showing lower modularity and average path length. Key species in the co‐occurrence network of *HA* included Woeseiaceae, Halomonadaceae, and unclassified_Rhizobiales among bacteria, and Phaeococcomycetaceae and Pleosporaceae among fungi. In contrast, Thermoanaerobaculaceae, Cyclobacteriaceae, and Glycomycetaceae bacteria, along with Didymosphaeriaceae, Cordycipitaceae, and Microascaceae fungi, were identified as keystone species in the network of *HP*.

**Figure 5:**
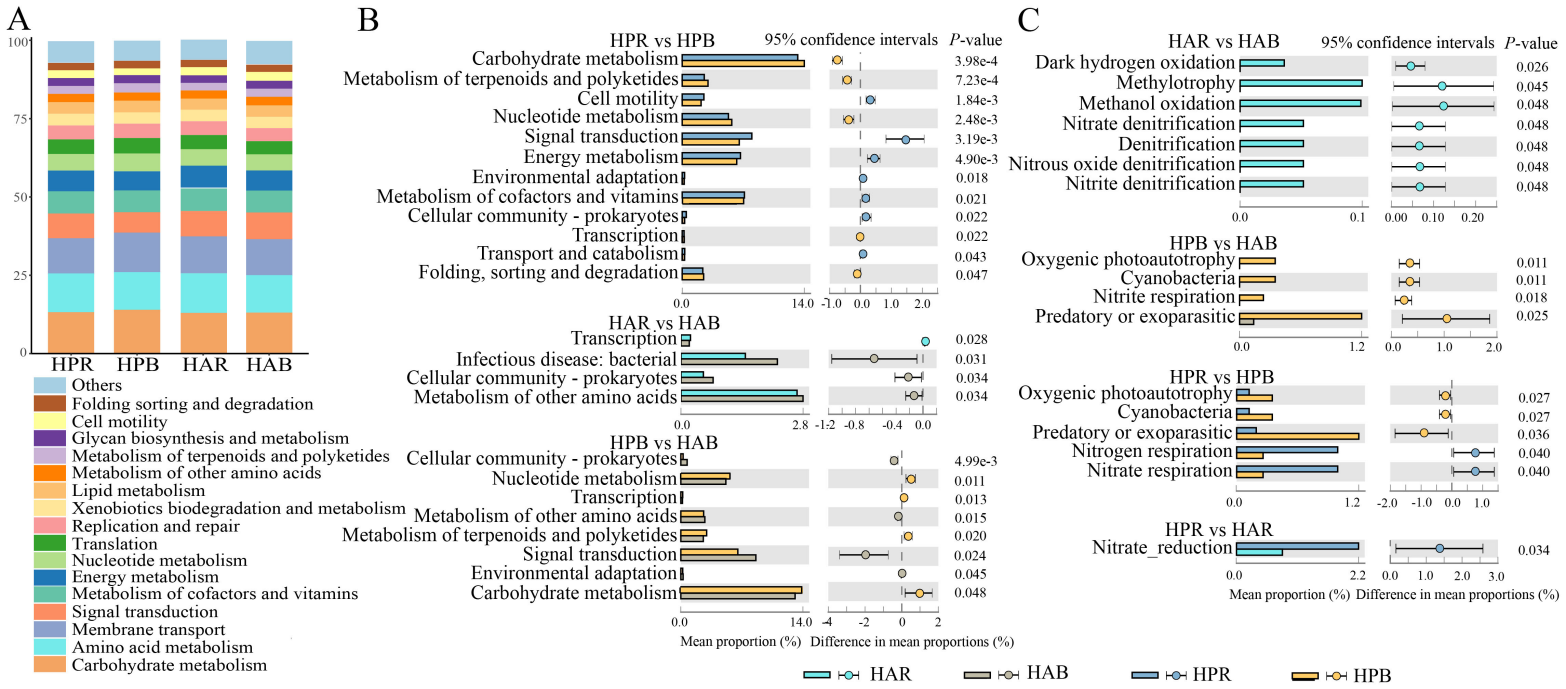
Function prediction and differential analysis of soil bacteria in the genus *Haloxylon.* **(A)** shows the composition of bacterial functional pathways at the KEGG class level, **(B)** presents the comparison of predicted functional pathways between groups, and **(C)** illustrates the difference in FAPROTAX functional prediction among groups.

The Tax4Fun analysis of KEGG pathways in soil bacteria of the genus *Haloxylon* revealed that carbohydrate and amino acid metabolism and membrane transport were the primary metabolic pathways (**Figure 5A**). Differential functional pathways were identified using the Wilcoxon test (*P*<0.05) (**Figure 5B**), highlighting significant differences in the following pathways: carbohydrate metabolism, signal transduction, metabolism of cofactors and vitamins, and energy metabolism in the soil bacteria of *HP*, with metabolism of other amino acids, infectious disease, and cellular community-prokaryotes of *HA*. Furthermore, carbohydrate metabolism, signal transduction, and nucleotide metabolism differed between the bulk soils of both species. The FAPROTAX database was utilized to predict differential functional processes in soil bacteria (*P*<0.05), with a focus on nitrogen and carbon cycle-related processes (**Figure 5C**).

**Figure 6:**
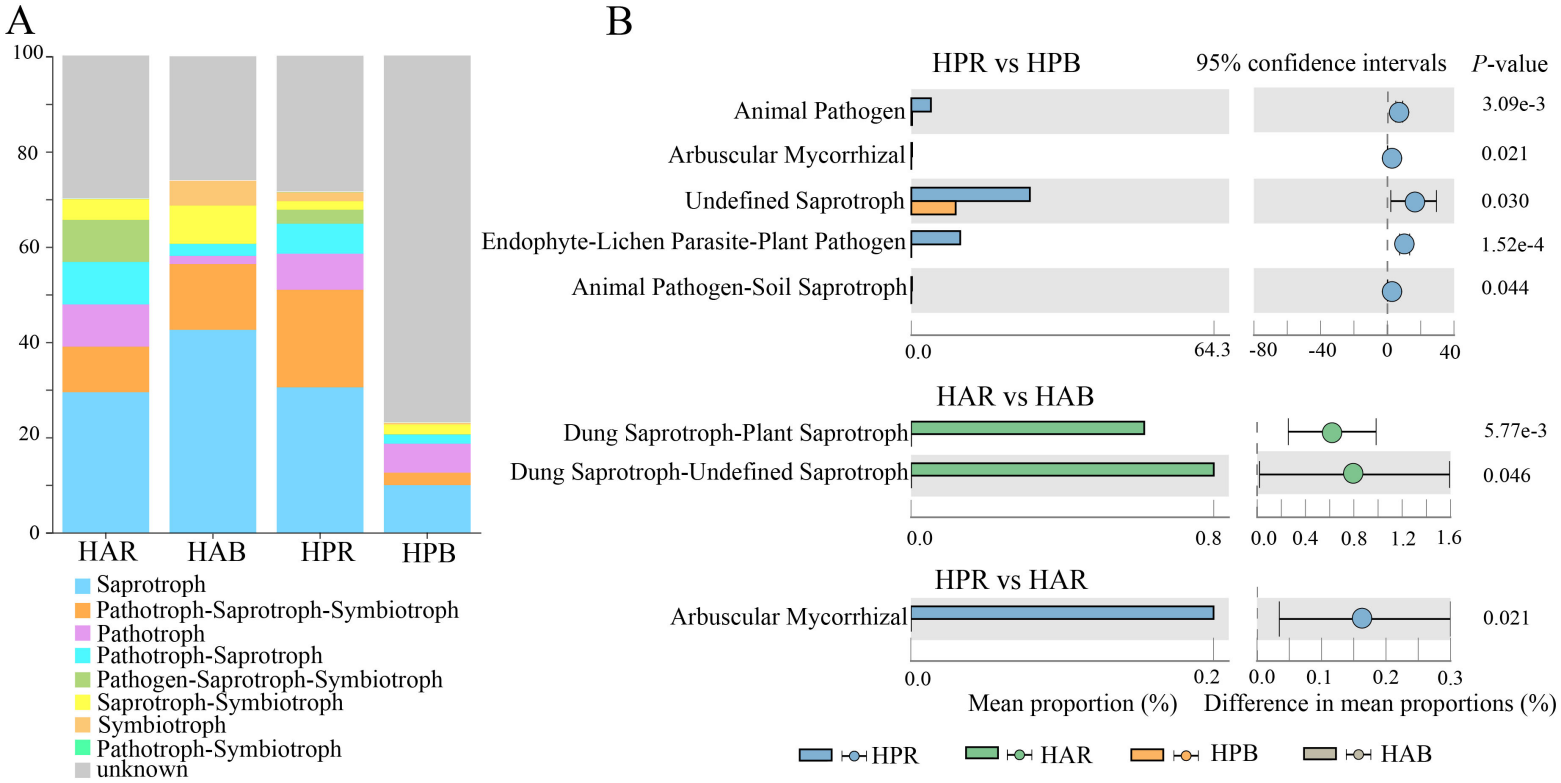
Prediction and differential analysis of fungal functional groups in the genus *Haloxylon*. **(A)** displays the relative abundance proportion of fungal functional prediction by FUNGuild. **(B)** presents the comparison of predicted functional pathways among groups.

We used FUNGuild function prediction and Wilcoxon test (*P*<0.05) to examine differences in soil fungal function within the genus *Haloxylon*. The primary functions identified for soil fungi in *Haloxylon* were Saprotroph and Sybiotrophs (**Figure 6A**). Significant differences were observed in the functional prediction of Undefined Saprotroph, Endophyte-Lichen Parasite-Plant Pathogen, and Arbuscular Mycorrhizal and Animal in soil fungi of *HP*, with Dung Saprotroph-Saprotroph in soil fungi of *HA*. Arbuscular Mycorrhiza function exhibited significant differences in rhizosphere soil fungi between the two species but not in bulk soil (**Figure 6B**).

### 3.4 Correlation analysis of root traits, exudates, and microorganisms of genus *Haloxylon*

**Figure 7:**
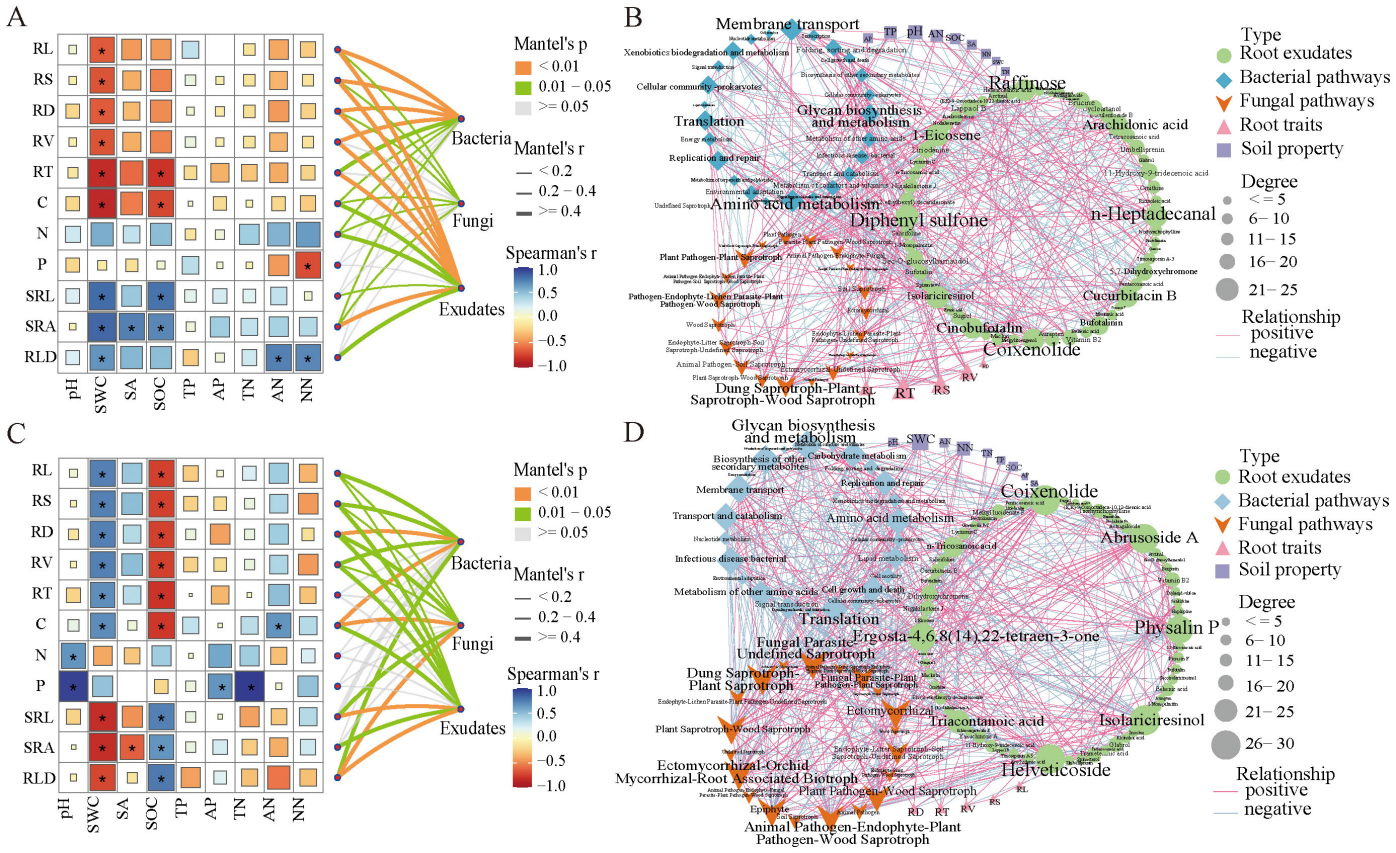
Correlation analysis between plant traits, soil environmental factors, and Mantel test of bacteria, fungi, and root exudates in the genus *Haloxylon*. **(A, C)** depict the Mantel test. **(B, D)** show the correlation network of bacteria, fungi, root exudates, soil properties, and root traits in the two *Haloxylon* species. **(A, B)** are for *HA*, while **(C, D)** represent *HP*. * indicated significant levels of *P* < 0.05.

At the family level, we conducted an analysis of the correlation between bacteria, fungi, root exudates, and root traits in the genus *Haloxylon* using the Mantel test. The RL, RS, RD, RV, and RT of the root trait of *HA* showed strong correlations with soil bacteria and root exudates but weak correlations with fungi (**Figure 7A**). Additionally, root traits were significantly correlated with SWC. In contrast, the RD, C, and RLD of the root traits of *HP* exhibited strong correlations with soil bacteria, fungi, and root exudates, and the root traits were significantly correlated with both SWC and SOC (**Figure 7C**).

Functional pathways prediction and Pearson’s correlation (|r| > 0.8, *P* < 0.05) revealed strong correlations between bacterial and fungal functions, root exudates, root traits, and soil properties (**Figures 7B, D**). Key hubs in the correlation network included root exudates such as Raffinose, n-heptadecanal, Coixenolide, and Diphenyl sulfone, with predicted microbial functions such as amino acid metabolism, membrane transport, glycan biosynthesis, and metabolism, dung saprotroph-plant saprotroph-wood saprotrophs, root traits T and S, and soil pH. The root exudates of *HP*, including isolariciresinol, abbrusoside A, helveticoside, physalin P, and coixenolide, along with predicted functional pathways, such as glycan biosynthesis and metabolism, biosynthesis of other secondary metabolites, membrane transport, dung saprotroph-plant saprotrophs, ectomycorrhizal function, root traits D and T, and soil SWC and NN, emerged as key hubs in the correlation network.

## 4 Discussion

### 4.1 Composition of root exudates within the genus *Haloxylon*

The quantity and quality of root exudates vary depending on factors such as plant genotype, soil environment, nutrient conditions, plant growth stage, and physiological state (Mommer et al., 2016). Root exudates from *HA* and *HP* plants exhibit significant differences in metabolite composition (**Figures 2A, B**), indicating variations in the components of the root exudates among different plant genotypes, consistent with previous studies (Ziegler et al., 2016). Plant‐derived secondary metabolites affect the complexity of interactions with biological and abiotic environmental factors (Enebe and Babalola, 2018). Terpenoids—a major chemical defense in plants—play an essential role in species competition and co-evolution (Fujii et al., 2012). The root exudates of *Haloxylon* exhibit significantly higher levels of triterpernoids than those of other compounds, with fatty acids and steroids following (**Figures 2C, D**). These may be adaptive strategies closely related species employ to thrive in desert and drought-stressed environments. Triterpenoids and fatty acids serve as signaling molecules, actively contributing to plant stress responses that support overall plant growth (Li et al., 2022b).

Stress affects protein synthesis and nucleic acid metabolism in plants, leading to increased secretion of organic acids and amino metabolites, such as oxalic acid and proline, during periods of stress (Canarini et al., 2016; Xiang et al., 2019). The metabolic pathways observed in the *Haloxylon* genus primarily include metabolic and organic system pathways, with significantly enriched pathways such as galactose metabolism, riboflavin metabolism, and arginine biosynthesis pathway. These pathways are closely linked to adaptation strategies for harsh desert environments, particularly in enhancing drought resistance through improved arginine and proline metabolism (Shi et al., 2013). The physiological and ecological response mechanisms of *Haloxylon* to environmental stress may slightly involve terpenoids and fatty acids as the primary differential metabolites in root exudates between the two species (**Figure 3**). The composition of root exudates is affected and constrained by factors such as plant species, genotype, stress conditions, and other environmental factors (Calvo et al., 2019; Vives-Peris et al., 2017).

### 4.2 Soil microbial communities and functional differences of the genus *Haloxylon*

Many Proteobacteria exhibit eutrophic characteristics, evidenced by rapid growth rates and the ability to utilize diverse substrates (Spain et al., 2009; Zhang et al., 2016). Microbial populations in various plant rhizosphere soils are generally affected by rhizosphere effects (Jing et al., 2023). At the phylum level, the bacterial genus of *Haloxylon* predominantly belonged to Proteobacteria and Actinobacteria, while fungi mainly belonged to Ascomycota and Basidiomycota (**Figures 4A, B**). Rhizosphere effect plays a vital role in shaping differences in microbial composition and diversity between rhizosphere soil and bulk soil (Qiu et al., 2022; Vieira et al., 2020). Rhizosphere available resources, specifically nutrients, are more abundant, while environmental stress is reduced (Lundberg et al., 2012; Qiu et al., 2022). The rhizosphere of *Haloxylon* shows higher microbial diversity than that of bulk soil, with a distinct composition that is significantly different (**Figures 4C**; **Supplementary Figure S3**). This indicates a strong selection of rhizosphere microbial community by two species, highlighting the effect of host plants on rhizosphere microbial community (Gao et al., 2020). The intricate relationships among plant hosts, microorganisms, and the environment strongly correlate with network complexity (Mougi and Kondoh, 2012; Tian et al., 2022). In the soil of the *Haloxylon* genus, the networks of bacteria and fungi exhibit similar levels of complexity (**Figures 4D, E**; **Supplementary Table S2**), while the key microbial species in these networks differ. Specifically, the rhizosphere of *HP* shows significant enrichment in Proteobacteria and Bacteroidota, while *HA* is primarily enriched in Bacteroidetes, Cyanobacteria, and Proteobacteria (**Supplementary Figure S4**). These differences likely stem from interactions between host plant genotypes and soil conditions, enabling adaption to diverse habitat conditions.

Metabolites produced by rhizosphere microbial communities play an important role in providing nutrients to plants or microorganisms, triggering and participating in plant responses to the environment, and enhancing plant stress tolerance (Bai et al., 2022; Sukweenadhi et al., 2015). Plants and microorganisms absorb ammonium salts, nitrates, and other inorganic forms of nitrogen from their environments to synthesize proteins and nitrogen compounds. In the rhizosphere of the *Haloxylon* genus, functional predictions of bacteria primarily revolve around nitrogen metabolism, showing significantly heightened activity than that of the bulk soil. These functions are mainly associated with nitrogen and carbon cycle processes (**Figure 5C**), indicating the close interaction between rhizosphere plants and soil microorganisms despite variations in the functional groups of rhizosphere microbial communities across different species. Environmental information processing pathways play a vital role in regulating various signaling pathways in bacteria, including responses to toxicity, changes in osmotic pressure, nutrient absorption, and secondary metabolite production (Ma et al., 2022). Similar patterns are observed in the genus *Haloxylon*, particularly focusing on metabolic and environmental information processing pathways, albeit with distinctions between the two species (**Figure 5B**). These differences likely stem from the physiological and ecological adaptations of plants to environmental stress, developed over long-term evolution, fostering symbiotic relationships with fungi that aid in regulating plant growth and enhancing resistance to harsh environments (Gill et al., 2016). The rhizosphere fungi of both species primarily exhibit saprophytic and saprophytic combinations traits, with higher functional diversity than that of bulk soil (**Figure 6**). This indicates that greater functional diversity in fungal species contributes to maintaining rhizosphere stability and overall plant health (Wagg et al., 2019).

### 4.3 Root exudates mediate synergistic interaction between soil properties, microorganisms and the root traits of the genus *Haloxylon*

During the growth phase of plants, roots secrete organic compounds into the surrounding soil environment. These compounds create and sustain a symbiotic relationship between plants and their soil environment, facilitating various biological and abiotic processes (Bais et al., 2006). Root traits are pivotal factors that affect nutrient and water absorption and are crucial for sustaining plant growth (Samejima et al., 2005; Zhang et al., 2009). Here, we found significant correlations between SWC and the root traits of *HA* and SWC, and SOC with the root traits of *HP* (**Figures 7A, C**). These correlations suggest that desert plants invest heavily in underground structures for water absorption to support their growth during drought periods. Moreover, the microbial communities in the root and rhizosphere also play a critical role. They contribute by supplying essential nutrients and water for plant growth while also participating in producing growth-regulating compounds, such as hormones, organic acids, and amino acids (Bai et al., 2022; Samejima et al., 2005). Strong correlations were observed between the root traits of *HA* and its exudates, soil bacteria, and between the root traits of *HP* and its exudates, soil bacteria, and fungi, indicating a close relationship between root traits and exudates (Meier et al., 2020; Sun et al., 2021; Weemstra et al., 2016). These correlations affect the composition and function of rhizosphere microorganisms (Bulgarelli et al., 2015; Zhalnina et al., 2018). The interactions between plants, soil, and rhizosphere bacteria and fungi mediated by root exudates differ between the two species and are affected by various factors such as soil properties, host plant phenotypes and traits, and abiotic environmental conditions (Berendsen et al., 2012; Vieira et al., 2020).

Roots secrete a range of primary metabolites and secondary metabolites, such as, carbohydrates, alkaloids, terpenes, and phenols (Enebe and Babalola, 2018). These compounds are crucial in shaping and affecting rhizosphere microbial communities and are closely linked to key rhizosphere processes such as carbon and nitrogen cycles (Bulgarelli et al., 2015; Sasse et al., 2018; Vives-Peris et al., 2020). Specifically, our findings reveal that root exudates from *Haloxylon* plants exhibit robust interactions with root traits, soil microbial functions, and soil properties (**Figures 7B, D**). The primary metabolites found in root exudates of *HA* include sugars and fatty acids (such as raffinose, n-heptadecanal, and coixenolide), with root traits T and S. Amino acid metabolism, membrane transport, and the soil microorganism pH are closely interconnected. *HP* relies on secondary metabolites, Steroids, and Terpenoids (such as helveticoside, ambrusoside A, and physalin P), with root traits T, and soil microorganisms, for glycan metabolism. The biosynthesis of other secondary metabolites, SWC and NN, exhibit strong mutual interactions, indicating distinct species‐specific interactions with their soil environment and microorganisms to enhance adaptability in stressed desert environments (De Vries et al., 2020; Quiza et al., 2015).

We conclude that the interaction between the genus *Haloxylon* growth, soil conditions and microorganisms are a crucial determinant for the exudation profiles observed. This study fills a gap in knowledge about the interactions between plant closely relative species and their subsurface rhizosphere ecological processes in desert ecosystems. However, exudates are dynamic, varying growth stages and season, and also differences in plant species. In this study, specific root exudate components and core microbial flora were not specifically screened, and the interaction between them was not comprehensive enough. This limitation may lead to a lack of depth and clarity in our understanding of how root exudations regulate plant-soil-microbial systems in response to adverse environmental changes. Therefore, in future studies, it is necessary to clarify the function of specific root exudates and coordinate the main beneficial microbial groups to resist stressed environments, so as to better reveal ecological adaptation strategies of plants under harsh environmental conditions and provide scientific basis for the conservation of *Haloxylon* in desert ecosystems and desertification control.

## 5 Conclusion

The profiles of root exudates in the genus Haloxylon are different, which mediates complex interactions between rhizosphere microorganisms and their hosts. However, these complex interactions manifest differently in the performance of the two species within the soil ecosystem. The findings highlight the significant role of root exudates in responding to soil environmental changes and shaping the structure of the rhizosphere microbial community, synergizing with the host plant, and ecological adaptation mechanisms of *Haloxylon* plants to environmental stressors and contribute to symbiotic relationships with soil microbes. Future studies can deepen our understanding of the complex interactions between desert plants, soil microbial communities, and the environment, ultimately contributing to strategies for enhancing plant resilience and ecosystem sustainability in arid regions.

## Data Availability

The original contributions presented in the study are included in the article/**Supplementary Material**. Further inquiries can be directed to the corresponding author.

## References

[B1] Baetz U. Martinoia E. (2014). Root exudates: the hidden part of plant defense. Trends Plant Sci. 19, 90–98. doi: 10.1016/j.tplants.2013.11.006 24332225

[B2] Bai B. Liu W. Qiu X. Zhang J. Zhang J. Bai Y. (2022). The root microbiome: Community assembly and its contributions to plant fitness. J. Integr. Plant Biol. 64, 230–243. doi: 10.1111/jipb.13226 35029016

[B3] Bais H. P. Weir T. L. Perry L. G. Gilroy S. Vivanco J. M. (2006). The role of root exudates in rhizosphere interactions with plants and other organisms. Annu. Rev. Plant Biol. 57, 233–266. doi: 10.1146/annurev.arplant.57.032905.105159 16669762

[B4] Berendsen R. L. Pieterse C. M. J. Bakker P. A. H. M. (2012). The rhizosphere microbiome and plant health. Trends Plant Sci. 17, 478–486. doi: 10.1016/j.tplants.2012.04.001 22564542

[B5] Bulgarelli D. Garrido-Oter R. Münch P. C. Weiman A. Dröge J. Pan Y.. (2015). Structure and function of the bacterial root microbiota in wild and domesticated barley. Cell Host Microbe 17, 392–403. doi: 10.1016/j.chom.2015.01.011 25732064 PMC4362959

[B6] Calvo O. C. Franzaring J. Schmid I. Fangmeier A. (2019). Root exudation of carbohydrates and cations from barley in response to drought and elevated CO2. Plant Soil 438, 127–142. doi: 10.1007/s11104-019-03998-y

[B7] Canarini A. Merchant A. Dijkstra F. A. (2016). Drought effects on Helianthus annuus and Glycine max metabolites: from phloem to root exudates. Rhizosphere 2, 85–97. doi: 10.1016/j.rhisph.2016.06.003

[B8] Chai Y. Cao Y. Yue M. Tian T. Yin Q. Dang H.. (2019). Soil abiotic properties and plant functional traits mediate associations between soil microbial and plant communities during a secondary forest succession on the loess plateau. Front. Microbiol. 10. doi: 10.3389/fmicb.2019.00895 PMC6499021 31105679

[B9] Christner B. C. Mosley-Thompson E. Thompson L. G. Reeve J. N. (2001). Isolation of bacteria and 16S rDNAs from Lake Vostok accretion ice. Environ. Microbiol. 3, 570–577. doi: 10.1046/j.1462-2920.2001.00226.x 11683867

[B10] Cui Y. Fang L. Guo X. Wang X. Wang Y. Li P.. (2018). Responses of soil microbial communities to nutrient limitation in the desert-grassland ecological transition zone. Sci. Total Environ. 642, 45–55. doi: 10.1016/j.scitotenv.2018.06.033 29894881

[B11] De La Fuente Cantó C. Simonin M. King E. Moulin L. Bennett M. J. Castrillo G.. (2020). An extended root phenotype: the rhizosphere, its formation and impacts on plant fitness. Plant J. 103, 951–964. doi: 10.1111/tpj.14781 32324287

[B12] De Vries F. T. Griffiths R. I. Knight C. G. Nicolitch O. Williams A. (2020). Harnessing rhizosphere microbiomes for drought-resilient crop production. Science 368, 270–274. doi: 10.1126/science.aaz5192 32299947

[B13] Enebe M. C. Babalola O. O. (2018). The influence of plant growth-promoting rhizobacteria in plant tolerance to abiotic stress: a survival strategy. Appl. Microbiol. Biotechnol. 102, 7821–7835. doi: 10.1007/s00253-018-9214-z 30030564 PMC6132541

[B14] Eppinga M. B. Rietkerk M. Dekker S. C. De Ruiter P. C. van der Putten W. H. (2006). Accumulation of local pathogens a new hypothesis to explain exotic plant invasions. Oikos 1, 168–176. doi: 10.1111/j.2006.0030-1299.14625.x

[B15] Fujii K. Aoki M. Kitayama K. (2012). Biodegradation of low molecular weight organic acids in rhizosphere soils from a tropical montane rain forest. Soil Biol. Biochem. 47, 142–148. doi: 10.1016/j.soilbio.2011.12.018

[B16] Gao C. Montoya L. Xu L. Madera M. Hollingsworth J. Purdom E.. (2020). Fungal community assembly in drought-stressed sorghum shows stochasticity, selection, and universal ecological dynamics. Nat. Commun. 11, 34. doi: 10.1038/s41467-019-13913-9 31911594 PMC6946711

[B17] Gardes M. Bruns T. D. (1993). ITS primers with enhanced specificity for basidiomycetes - application to the identification of mycorrhizae and rusts. Mol. Ecol. 2, 113–118. doi: 10.1111/j.1365-294X.1993.tb00005.x 8180733

[B18] Gill S. S. Gill R. Trivedi D. K. Anjum N. A. Sharma K. K. Ansari M. W.. (2016). Piriformospora indica: potential and significance in plant stress tolerance. Front. Microbiol. 7. doi: 10.3389/fmicb.2016.00332 PMC4801890 27047458

[B19] Haichar F. E. Z. Santaella C. Heulin T. Achouak W. (2014). Root exudates mediated interactions belowground. Soil Biol. Biochem. 77, 69–80. doi: 10.1016/j.soilbio.2014.06.017

[B20] Harbort C. J. Hashimoto M. Inoue H. Niu Y. Guan R. Rombolà A. D. (2020). Root-secreted coumarins and the microbiota interact to improve iron nutrition in arabidopsis. Cell Host Microbe 28, 825–837.e6. doi: 10.1016/j.chom.2020.09.006 33027611 PMC7738756

[B21] Jakoby G. Rog I. Megidish S. Klein T. (2020). Enhanced root exudation of mature broadleaf and conifer trees in a Mediterranean forest during the dry season. Tree Physiol. 40, 1595–1605. doi: 10.1093/treephys/tpaa092 32705136

[B22] Jing H. Wang H. Wang G. Liu G. Cheng Y. (2023). The mechanism effects of root exudate on microbial community of rhizosphere soil of tree, shrub, and grass in forest ecosystem under N deposition. ISME Commun. 3, 120. doi: 10.1038/s43705-023-00322-9 37985715 PMC10662252

[B23] Kardol P. Cornips N. J. van Kempen M. M. L. Bakx-Schotman J. M. T. van der Putten W. H. (2007). Microbe-mediated plant-soil feedback causes historical contingency effects in plant community assembly. Ecol. Monogr. 77, 147–162. doi: 10.1890/06-0502

[B24] Korenblum E. Dong Y. Szymanski J. Panda S. Jozwiak A. Massalha H.. (2020). Rhizosphere microbiome mediates systemic root metabolite exudation by root-to-root signaling. Proc. Natl. Acad. Sci. 117, 3874–3883. doi: 10.1073/pnas.1912130117 32015118 PMC7035606

[B25] Lareen A. Burton F. Schäfer P. (2016). Plant root-microbe communication in shaping root microbiomes. Plant Mol. Biol. 90, 575–587. doi: 10.1007/s11103-015-0417-8 26729479 PMC4819777

[B26] Li W. Li Y. Lv J. He X. Wang J. Teng D.. (2022a). Rhizosphere effect alters the soil microbiome composition and C, N transformation in an arid ecosystem. Appl. Soil Ecol. 170, 104296. doi: 10.1016/j.apsoil.2021.104296

[B27] Li Y. Zou J. Zhu H. He J. Setter T. L. Wang Y.. (2022b). Drought deteriorated the nutritional quality of cottonseed by altering fatty acids and amino acids compositions in cultivars with contrasting drought sensitivity. Environ. Exp. Bot. 194, 104747. doi: 10.1016/j.envexpbot.2021.104747

[B28] Lundberg D. S. Lebeis S. L. Paredes S. H. Yourstone S. Gehring J. Malfatti S.. (2012). Defining the core Arabidopsis thaliana root microbiome. Nature 488, 86–90. doi: 10.1038/nature11237 22859206 PMC4074413

[B29] Ma S. Qiao L. Liu X. Zhang S. Zhang L. Qiu Z.. (2022). Microbial community succession in soils under long-term heavy metal stress from community diversity-structure to KEGG function pathways. Environ. Res. 214, 113822. doi: 10.1016/j.envres.2022.113822 35803340

[B30] Meier I. C. Tückmantel T. Heitkötter J. Müller K. Preusser S. Wrobel T. J.. (2020). Root exudation of mature beech forests across a nutrient availability gradient: the role of root morphology and fungal activity. New Phytol. 226, 583–594. doi: 10.1111/nph.16389 31868933

[B31] Mommer L. Kirkegaard J. Van Ruijven J. (2016). Root–root interactions: towards A rhizosphere framework. Trends Plant Sci. 21, 209–217. doi: 10.1016/j.tplants.2016.01.009 26832947

[B32] Morgan J. A. W. Bending G. D. White P. J. (2005). Biological costs and benefits to plant–microbe interactions in the rhizosphere. J. Exp. Bot. 56, 1729–1739. doi: 10.1093/jxb/eri205 15911554

[B33] Mougi A. Kondoh M. (2012). Diversity of interaction types and ecological community stability. Science 337, 349–351. doi: 10.1126/science.1220529 22822151

[B34] Phillips R. P. Erlitz Y. Bier R. Bernhardt E. S. (2008). New approach for capturing soluble root exudates in forest soils. Funct. Ecol. 22, 990–999. doi: 10.1111/j.1365-2435.2008.01495.x

[B35] Qiu L. Kong W. Zhu H. Zhang Q. Banerjee S. Ishii S.. (2022). Halophytes increase rhizosphere microbial diversity, network complexity and function in inland saline ecosystem. Sci. Total Environ. 831, 154944. doi: 10.1016/j.scitotenv.2022.154944 35367547

[B36] Quiza L. St-Arnaud M. Yergeau E. (2015). Harnessing phytomicrobiome signaling for rhizosphere microbiome engineering. Front. Plant Sci. 6. doi: 10.3389/fpls.2015.00507 PMC4500914 26236319

[B37] Ribbons R. R. Levy-Booth D. J. Masse J. Grayston S. J. McDonald M. A. Vesterdal L.. (2016). Linking microbial communities, functional genes and nitrogen-cycling processes in forest floors under four tree species. Soil Biol. Biochem. 103, 181–191. doi: 10.1016/j.soilbio.2016.07.024

[B38] Sahu P. K. Singh D. P. Prabha R. Meena K. K. Abhilash P. C. (2019). Connecting microbial capabilities with the soil and plant health: Options for agricultural sustainability. Ecol. Indic. 105, 601–612. doi: 10.1016/j.ecolind.2018.05.084

[B39] Samejima H. Kondo M. Ito O. Nozoe T. Shinano T. Osaki M. (2005). Characterization of root systems with respect to morphological traits and nitrogen-absorbing ability in the new plant type of tropical rice lines. J. Plant Nutr. 28, 835–850. doi: 10.1081/PLN-200055550

[B40] Sasse J. Martinoia E. Northen T. (2018). Feed your friends: do plant exudates shape the root microbiome? Trends Plant Sci. 23, 25–41. doi: 10.1016/j.tplants.2017.09.003 29050989

[B41] Semchenko M. Saar S. Lepik A. (2014). Plant root exudates mediate neighbour recognition and trigger complex behavioural changes. New Phytol. 204, 631–637. doi: 10.1111/nph.12930 25039372

[B42] Shi H. Ye T. Chen F. Cheng Z. Wang Y. Yang P.. (2013). Manipulation of arginase expression modulates abiotic stress tolerance in Arabidopsis: effect on arginine metabolism and ROS accumulation. J. Exp. Bot. 64, 1367–1379. doi: 10.1093/jxb/ers400 23378380 PMC3598423

[B43] Song J. Feng G. Tian C.-Y. Zhang F.-S. (2006). Osmotic adjustment traits of Suaeda physophora, Haloxylon ammodendron and Haloxylon persicum in field or controlled conditions. Plant Sci. 170, 113–119. doi: 10.1016/j.plantsci.2005.08.004

[B44] Spain A. M. Krumholz L. R. Elshahed M. S. (2009). Abundance, composition, diversity and novelty of soil *Proteobacteria* . ISME J. 3, 992–1000. doi: 10.1038/ismej.2009.43 19404326

[B45] Sukweenadhi J. Kim Y.-J. Choi E.-S. Koh S.-C. Lee S.-W. Kim Y.-J.. (2015). Paenibacillus yonginensis DCY84T induces changes in Arabidopsis thaliana gene expression against aluminum, drought, and salt stress. Microbiol. Res. 172, 7–15. doi: 10.1016/j.micres.2015.01.007 25721473

[B46] Sun L. Ataka M. Han M. Han Y. Gan D. Xu T.. (2021). Root exudation as a major competitive fine-root functional trait of 18 coexisting species in a subtropical forest. New Phytol. 229, 259–271. doi: 10.1111/nph.16865 32772392

[B47] Tian G. Qiu H. Li D. Wang Y. Zhen B. Li H.. (2022). Little environmental adaptation and high stability of bacterial communities in rhizosphere rather than bulk soils in rice fields. Appl. Soil Ecol. 169, 104183. doi: 10.1016/j.apsoil.2021.104183

[B48] Venturi V. Keel C. (2016). Signaling in the rhizosphere. Trends Plant Sci. 21, 187–198. doi: 10.1016/j.tplants.2016.01.005 26832945

[B49] Vieira S. Sikorski J. Dietz S. Herz K. Schrumpf M. Bruelheide H.. (2020). Drivers of the composition of active rhizosphere bacterial communities in temperate grasslands. ISME J. 14, 463–475. doi: 10.1038/s41396-019-0543-4 31659233 PMC6976627

[B50] Vives-Peris V. De Ollas C. Gómez-Cadenas A. Pérez-Clemente R. M. (2020). Root exudates: from plant to rhizosphere and beyond. Plant Cell Rep. 39, 3–17. doi: 10.1007/s00299-019-02447-5 31346716

[B51] Vives-Peris V. Gómez-Cadenas A. Pérez-Clemente R. M. (2017). Citrus plants exude proline and phytohormones under abiotic stress conditions. Plant Cell Rep. 36, 1971–1984. doi: 10.1007/s00299-017-2214-0 29038909

[B52] Wagg C. Schlaeppi K. Banerjee S. Kuramae E. E. van der Heijden M. G. A. (2019). Fungal-bacterial diversity and microbiome complexity predict ecosystem functioning. Nat. Commun. 10, 4841. doi: 10.1038/s41467-019-12798-y 31649246 PMC6813331

[B53] Wang H. Zhang R. Cai Y. Yang Q. Lv G. (2022). Ecological uniqueness and the determinants in arid desert ecosystems of Northwest China. Glob. Ecol. Conserv. 34, e02005. doi: 10.1016/j.gecco.2022.e02005

[B54] Weemstra M. Mommer L. Visser E. J. W. Van Ruijven J. Kuyper T. W. Mohren G. M. J.. (2016). Towards a multidimensional root trait framework: a tree root review. New Phytol. 211, 1159–1169. doi: 10.1111/nph.14003 27174359

[B55] Wen Z. Li H. Shen Q. Tang X. Xiong C. Li H.. (2019). Tradeoffs among root morphology, exudation and mycorrhizal symbioses for phosphorus-acquisition strategies of 16 crop species. New Phytol. 223, 882–895. doi: 10.1111/nph.15833 30932187

[B56] Xia Z. Yu L. He Y. Korpelainen H. Li C. (2019). Broadleaf trees mediate chemically the growth of Chinese fir through root exudates. Biol. Fertil Soils 55, 737–749. doi: 10.1007/s00374-019-01389-0

[B57] Xiang G. Ma W. Gao S. Jin Z. Yue Q. Yao Y. (2019). Transcriptomic and phosphoproteomic profiling and metabolite analyses reveal the mechanism of NaHCO3-induced organic acid secretion in grapevine roots. BMC Plant Biol. 19, 383. doi: 10.1186/s12870-019-1990-9 31481025 PMC6724372

[B58] Yin H. Li Y. Xiao J. Xu Z. Cheng X. Liu Q. (2013). Enhanced root exudation stimulates soil nitrogen transformations in a subalpine coniferous forest under experimental warming. Glob. Change Biol. 19, 2158–2167. doi: 10.1111/gcb.12161 23504744

[B59] Zhalnina K. Louie K. B. Hao Z. Mansoori N. Da Rocha U. N. Shi S.. (2018). Dynamic root exudate chemistry and microbial substrate preferences drive patterns in rhizosphere microbial community assembly. Nat. Microbiol. 3, 470–480. doi: 10.1038/s41564-018-0129-3 29556109

[B60] Zhang C. Liu G. Xue S. Wang G. (2016). Soil bacterial community dynamics reflect changes in plant community and soil properties during the secondary succession of abandoned farmland in the Loess Plateau. Soil Biol. Biochem. 97, 40–49. doi: 10.1016/j.soilbio.2016.02.013

[B61] Zhang H. Xue Y. Wang Z. Yang J. Zhang J. (2009). An alternate wetting and moderate soil drying regime improves root and shoot growth in rice. Crop Sci. 49, 2246–2260. doi: 10.2135/cropsci2009.02.0099

[B62] Zhao M. Zhao J. Yuan J. Hale L. Wen T. Huang Q.. (2021). Root exudates drive soil‐microbe‐nutrient feedbacks in response to plant growth. Plant Cell Environ. 44, 613–628. doi: 10.1111/pce.13928 33103781

[B63] Zhu Y. Zhang S. Huang H. Wen B. (2009). Effects of maize root exudates and organic acids on the desorption of phenanthrene from soils. J. Environ. Sci. 21, 920–926. doi: 10.1016/S1001-0742(08)62362-1 19862957

[B64] Ziegler J. Schmidt S. Chutia R. Müller J. Böttcher C. Strehmel N.. (2016). Non-targeted profiling of semi-polar metabolites in Arabidopsis root exudates uncovers a role for coumarin secretion and lignification during the local response to phosphate limitation. J. Exp. Bot. 67, 1421–1432. doi: 10.1093/jxb/erv539 26685189 PMC4762384

