# Supplementary material for: Root exudates facilitate the regulation of soil microbial community function in the genus Haloxylon
Source: Front Plant Sci. 2024 Sep 19;15:1461893. doi: 10.3389/fpls.2024.1461893 (PMC11446799; doi:10.3389/fpls.2024.1461893)
Supplement: Supplementary file 1 [file DataSheet1.docx]

**Supplementary material**

# Supplementary Figures and Tables

1.1 Supplementary Tables

Supplementary Table 1

Determination methods and main instruments employed for sample analysis

| No | Indicators | Abbreviation | Determination method | Main instrument |
| --- | --- | --- | --- | --- |
| 1 | Soil moisture content | SOW | Weight method | Electronic balance, HZK-FA110, Shanghai |
| 2 | Soil salinity content | SA |  |  |
| 3 | pH | / | Potentiometry | Leici PHS-3C, Shanghai Yidian Scientific Instrument |
| 4 | Soil organic carbon/  plant carbon | SOC/C | Potassium dichromate dilution heat method | / |
| 5 | Soil total nitrogen/  plant nitrogen | TN/N | Kjeldahl method | BUCHI-K370, BUCHI Labortechnik AG, Switzerlan |
| 6 | Soil ammonium nitrogen | AN | Indophenol blue colorimetry | Spectrophotometer UV1200 |
| 7 | Soil nitrate nitrogen | NN | Colorimetry of phenoldisulfonic acid | Spectrophotometer UV1200 |
| 8 | Soil total phosphorus/  plant phosphorus | TP/P | Molybdenum antimony anticolorimetric method | Spectrophotometer UV1200 |
| 9 | Soil available phosphorus | AP | Molybdenum antimony anticolorimetric method | Spectrophotometer UV1200 |
| 10 | Root length | L | Scanning | WinRHIZO, Regent Instruments Inc., Quebec, Canada |
| 11 | Root surface  Area | S |  |  |
| 12 | Root Volume | V |  |  |
| 13 | Root average diameter | D |  |  |
| 14 | Root tips | T |  |  |

Supplementary Table 2

Topological properties of microbial networks of *the genus Haloxylon*.

| Species | Number of families | |  | Edge | | | Average degree | Gram  density | Modularity | Average clustering coefficient | Average path length |
| --- | --- | --- | --- | --- | --- | --- | --- | --- | --- | --- | --- |
|  | Bacteria | Fungi |  | Bacteria-Bacteria | Bacteria-Fungi | Fungi-Fungi |  |  |  |  |  |
| *HA* | 406 | 112 |  | 74.41% | 23.24% | 2.35% | 15.814 | 0.186 | 0.391 | 0.616 | 2.318 |
| *HP* | 416 | 196 |  | 73.87% | 23.28% | 2.85% | 9.545 | 0.110 | 0.437 | 0.545 | 3.378 |

Supplementary Table 3

List of acronyms in the manuscript

| Abbreviation | Full name |
| --- | --- |
| SWC | Soil water content |
| SA | Soil salt content |
| SOC | Soil organic carbon |
| TP | Soil total phosphorus |
| AP | Soil available phosphorus |
| TN | Soil total nitrogen |
| AN | Soil ammonium nitrogen |
| NN | Soil nitrate nitrogen |
| RL | Root length |
| RS | Root surface area |
| RV | Root volume |
| RD | Root mean diameter |
| RT | Number of root tip |
| RM | Root mass |
| SRL | Specific root length |
| SRA | Specific root surface area |
| RLD | Root tissue density |
| C | Plant carbon content |
| N | Plant nitrogen content |
| P | Plant phosphorus content |
| HA | *Haloxylon ammodendron* |
| HP | *Haloxylon persicum* |
| HAR | Rhizosphere soil of *Haloxylon ammodendron* |
| HAB | Bulk soil of *Haloxylon ammodendron* |
| HPR | Rhizosphere soil of *Haloxylon persicum* |
| HPB | Bulk soil of *Haloxylon persicum* |

**1.2 Supplementary Figures**


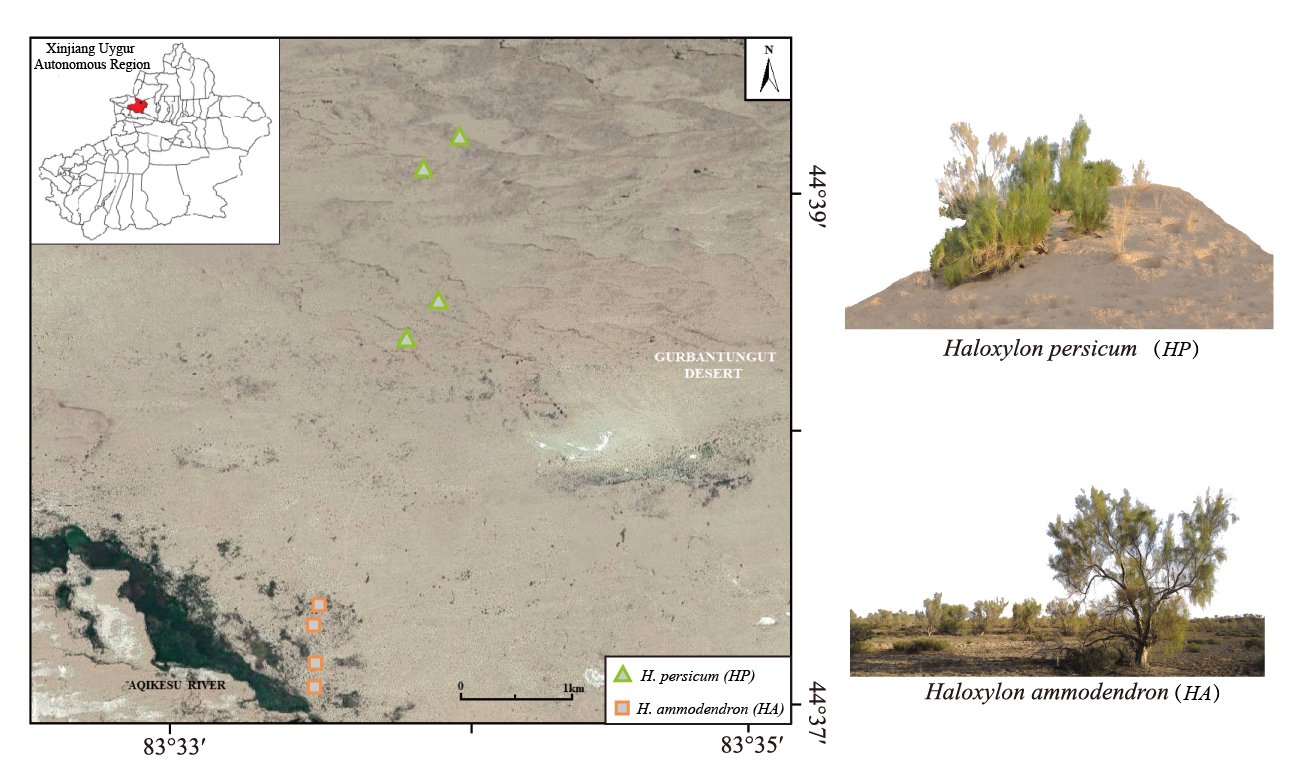


Fig. 1. The study area and location of the plots


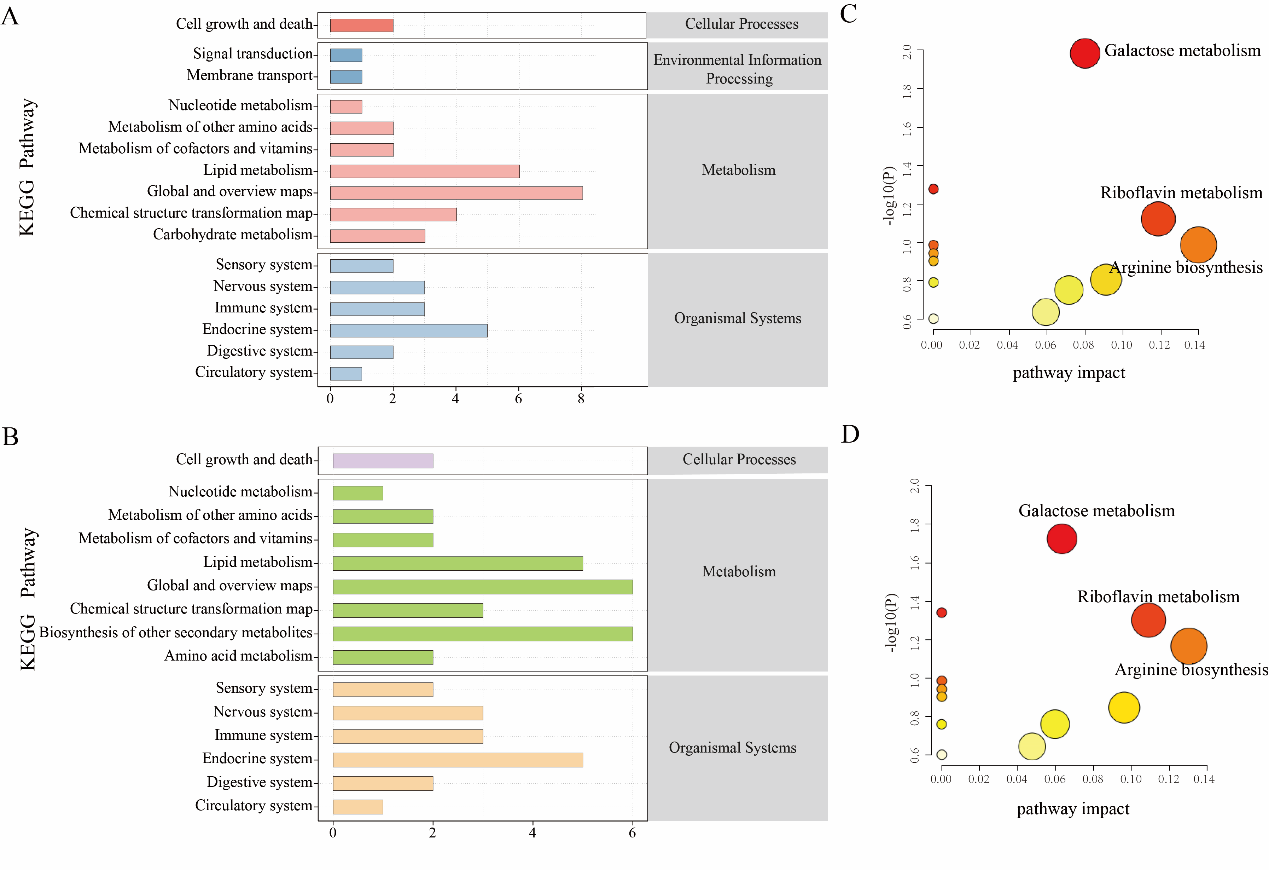


Supplementary Figure 2

Annotated and enrichment pathways of root exudates in the KEGG database. A and C represent *H. ammodendron*. B and D depict *H. persicum.*


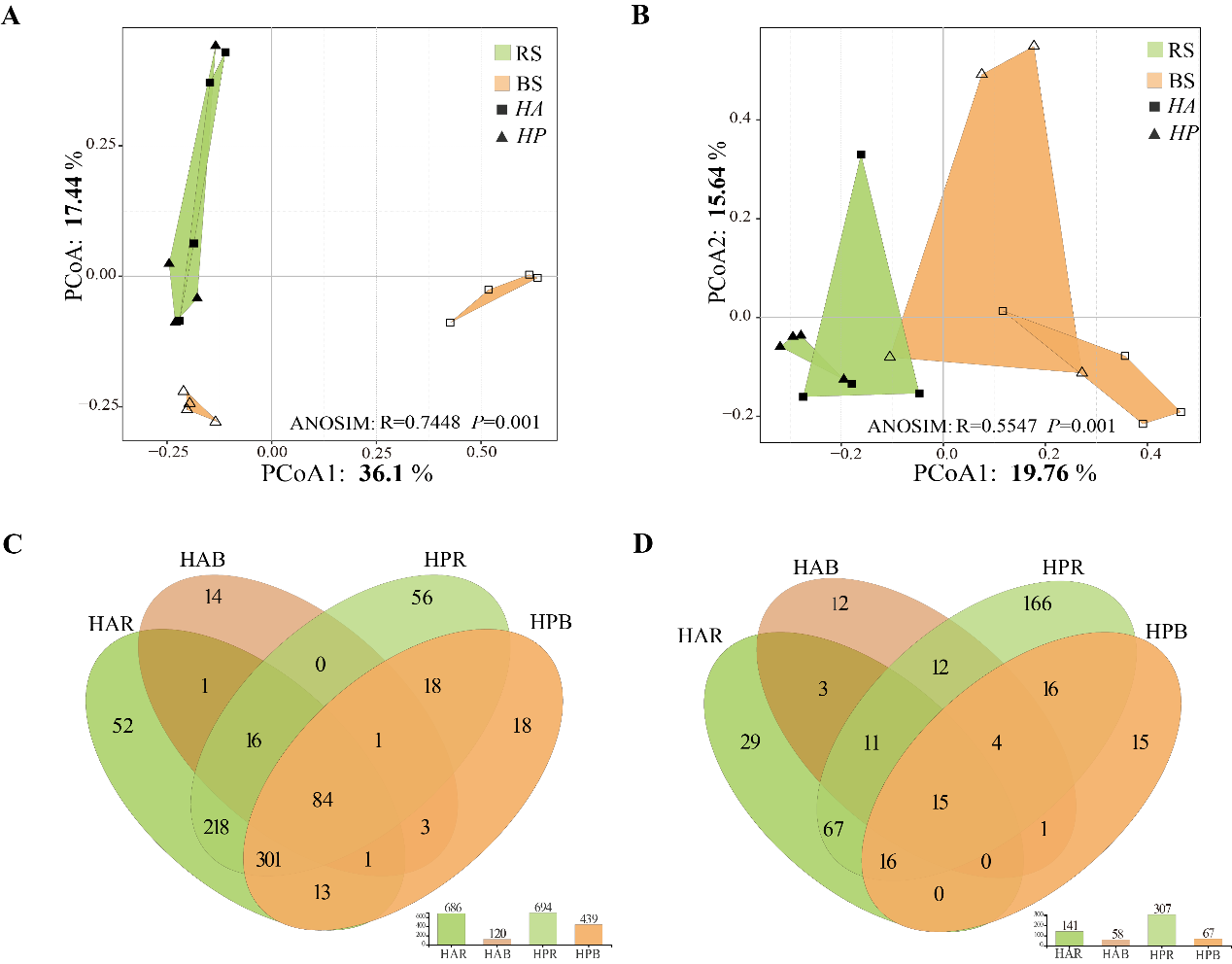


Supplementary Figure 3

Comparison of soil microbial community similarities using PCoA based on Bray–Curtis dissimilarities for four soil types: bacteria (A) and fungi (B). The results of the similarity analysis (ANOSIM) are shown at the top of A and B. Soil types are delineated using a 97% similarity distance, represented by a Venn diagram for bacteria (C) and Fungi (D).


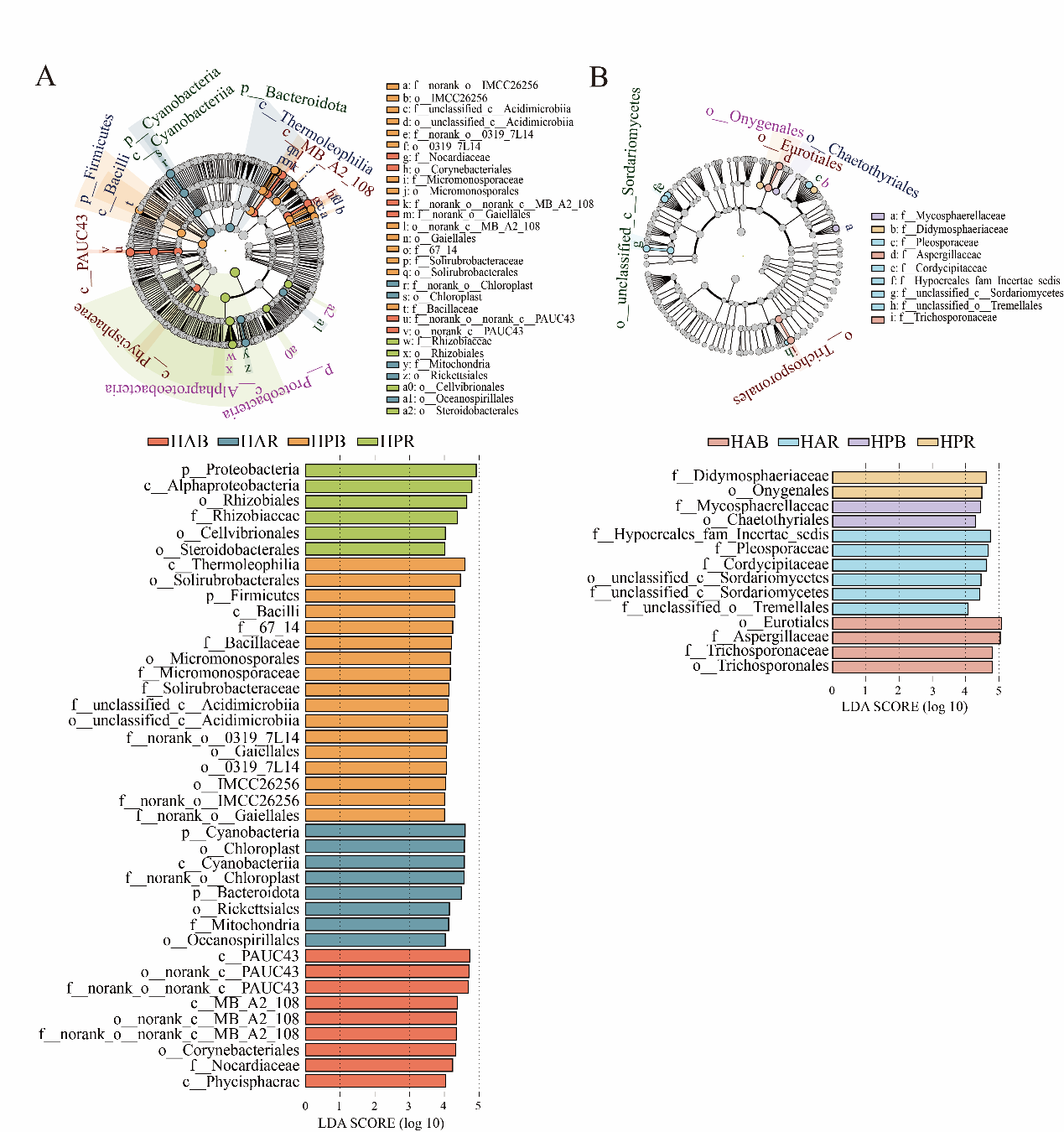


Supplementary Figure 4

Differential enrichment analysis of soil bacteria and fungi in the *genus Haloxylon*. A and C present the LEFSe differential enrichment analysis for the soil bacteria, and B and D depict the LEFSe differential enrichment analysis for soil fungi of the genus *Haloxylon,* LDA＞4. HAR denotes the rhizosphere soil, while HAB indicates the bulk soil of *H. ammodendron*; HPR refers to rhizosphere soil, and HPB represents the bulk soil of *H. Persicum*.
